# Supplementary material for: In Vitro Analysis of the Antagonistic Biological and Chemical Interactions between the Endophyte Sordaria tomento-alba and the Phytopathogen Botrytis cinerea
Source: Int J Mol Sci. 2024 Jan 13;25(2):1022. doi: 10.3390/ijms25021022 (PMC10816056; doi:10.3390/ijms25021022)
Supplement: Supplementary file 1 [file ijms-25-01022-s001.zip › ijms-2795276-supplementary.pdf]

Supplementary Materials

In vitro analysis of the antagonistic biological and chemical interactions between the endophyte *Sordaria tomento-alba* and the phytopathogen *Botrytis cinerea*

Hernando José Bolívar-Anillo[2,3†], Inmaculada Izquierdo-Bueno[1,2†], Estrella González-Rey[2], Victoria E. González-Rodríguez[1], Jesús M. Cantoral[1], Isidro G. Collado[2*] and Carlos Garrido[1*]

[1] Departamento de Biomedicina, Biotecnología y Salud Pública, Laboratorio de Microbiología, Facultad de Ciencias del Mar y Ambientales, Universidad de Cádiz, 11510, Puerto Real, Spain.
[2] Departamento de Química Orgánica, Facultad de Ciencias, Campus Universitario Río San Pedro s/n, Torre sur, 4ª planta, Universidad de Cádiz, 11510, Puerto Real, Cádiz, Spain.
[3] Programa de Microbiología, Facultad de Ciencias Básicas y Biomédicas, Universidad Simón Bolívar, Barranquilla, Colombia.
† Hernando José Bolívar-Anillo and Inmaculada Izquierdo-Bueno have contributed equally to this work
* Correspondence: Isidro G. Collado, isidro.gonzalez@uca.es; Carlos Garrido, carlos.garrido@uca.es

Table of contents:

Spectroscopic constant Tables S1-S7…………...……………….…………...**S2-S8**

Table S1. NMR Spectroscopic data of *trans*-sordariol (1) (^{1}H at 500 MHz, ^{13}C at 125 MHz, CD_3OD).

Position	Proton	δ_H (Hz)	$\delta^{13}C$	HMBC
1	H-1	-	125.2	
2	H-2	-	157.3	
3	H-3	6.7 (1H, dd, *J* = 8.0, 1.2 Hz)	115.4	C1, C2, C5
4	H-4	7.06 (1H, t, *J* = 8.0 Hz)	129.7	C2, C6
5	H-5	7.0 (1H, dd, *J* = 8.0, 1,2 Hz)	118.7	C1, C3, C4, C6
6	H-6	-	139.7	
7	H-7	4.8 (1H, s)	56.6	C1, C2, C6
1′	H-1′	6.98 (1H, d, *J* = 15.7, 1.0 Hz)	130.6	C1, C3′, C5, C6
2′	H-2′	6.17 (1H, dd, *J* = 15.7, 6.6 Hz)	132.6	C3′, C4′, C6
3′	H-3′	4.1 (1H, ddd, *J* = 6.6, 4.9, 1.3 Hz)	78.0	C1′, C2′, C4′, C5′
4′	H-4′	3.8 (1H, qd, *J* = 6.4, 4.9 Hz)	71.7	C2′, C3′, C5′
5′	H-5′	1.2 (3H, d, *J* = 6.4 Hz)	18.9	C3′, C4′

Table S2. NMR Spectroscopic data of *trans*-sordarial (2) (^{1}H at 500 MHz, ^{13}C at 125 MHz, $CDCl_3$).

Position	Proton	δ_H (Hz)	δ^{13}C	HMBC
1	H-1	-	117.3	
2	H-2	-	162.8	
3	H-3	6.9 (1H, dt, *J* = 8.4, 0.9 Hz)	117.3	C1, C2, C5
4	H-4	7.46 (1H, dd *J* = 8.4, 7.7 Hz)	137.2	C2, C6
5	H-5	6.94 (1H, dt, *J* = 7.7, 0.9 Hz	118.9	C1, C3, C4, C1´
6	H-6	-	142.1	
7	H-7	10.3 (1H, s)	195.2	C1, C2
1´	H-1´	7.2 (1H, dt, *J* = 15.7, 1.0 Hz)	126.9	C1, C2´,C3´, C5, C6
2´	H-2´	6.2 (1H, dd, *J* = 15.7, 6.1 Hz)	134.8	C1´, C3´, C4´, C6
3´	H-3´	4.3 (1H, ddd, *J* = 6.1, 3.5, 1.5 Hz)	75.8	C1´, C2´, C4´
4´	H-4´	4.0 (1H, qd, *J* = 6.5, 3.5 Hz)	70.2	C2´
5´	H-5´	1.2 (3H, d, *J* = 6.5 Hz)	17.7	C3´, C4´

Table S3. NMR Spectroscopic data of 3´-episordariol (3) (^{1}H at 500 MHz, ^{13}C at 125 MHz, ($CDCl_3$).

Position	Proton	δ_H (Hz)	δ^{13}C	HMBC
1	H-1	-	121.8	
2	H-2	-	156.5	
3	H-3	6.83 (1H, d(superimposed), *J* = 7.9 Hz)	116.2	C2, C5
4	H-4	7.16 (1H, t, *J* = 7.8 Hz)	129.1	C2, C6
5	H-5	6.94 (1H, dd, *J* = 7.8, 1.1 Hz	118.9	C1, C3, C4
6	H-6	-	136.2	
7	H-7	5.0 (1H, s)	60.2	
1´	H-1´	6.85 (1H, d, *J* = 15.7 Hz)	129.6	C3´, C5
2´	H-2´	6.1 (1H, dd, *J* = 15.7, 6.6 Hz)	131.0	C-6
3´	H-3´	4.3 (1H, s(br))	76.2	
4´	H-4´	4.0 (1H, dd(br), *J* = 6.4, 3.6 Hz)	70.3	C2´
5´	H-5´	1.2 (3H, d, *J* = 6.5 Hz)	17.7	C3´, C4´

Table S4. NMR Spectroscopic data of heptacyclosordariolone (4) (^{1}H at 500 MHz, ^{13}C at 125 MHz, Cl_3CD).

Position	Proton	δ_H (Hz)	$\delta^{13}C$	HMBC
1	H-1	-	126.7	
2	H-2	-	151.8	
3	H-3	6.7 (1H, dd, *J* = 7.7, 0.8 Hz)	131.0	C5
4	H-4	7.13 (1H, t, *J* = 7.7 Hz)	127.9	C2, C6
5	H-5	6.9 (1H, dd, *J* = 7.7, 0.8 Hz	123.4	C1, C3, C4, C1′
6	H-6	-	137.1	
7	H-7a	4.64 (1H, d, *J* = 14 Hz)	56.7	C1, C2, C6, C3′
	H-7b	5.1 (1H, d, *J* = 14 Hz)		C1, C2, C6, C3′
1′	H-1′	6.7 (1H, d, *J* = 12.5 Hz)	114.6	C1, C2′,C3′, C5
2′	H-2′	5.97 (1H, d, *J* = 12.5 Hz)	130.9	C3′, C6
3′	H-3′	-	104.0	
4′	H-4′	4.15 (1H, q, *J* = 6.5 Hz)	66.9	C5′, C3′, C2′
5′	H-5′	1.1 (3H, d, *J* = 6.5 Hz)	16.1	C3′, C4′
C2-OH				C1, C2

Table S5. NMR Spectroscopic data of cyclosordariolone (5) (^{1}H at 400 MHz, ^{13}C at 100 MHz, CD_3OD).

Position	Proton	δ_H (Hz)	$\delta^{13}C$	HMBC
1	H-1	-	126.3	
2	H-2	-	156.6	
3	H-3	6.91 (1H, d, *J* =8.5 Hz)	118.3	C1, C5
4	H-4	7.47 (1H, d, *J* =8.5 Hz)	127.3	C2, C6, C4′
5	H-5	-	139.0	
6	H-6	-	129.9	
7	H-7a	4.9 (1H, d, *J* = 11.8 Hz)	55.0	C1, C2, C6
	H-7b	4.84 (1H, d, *J* = 11.8 Hz)		
1′	H-1′	7.97 (1H, d, *J* = 10.3 Hz)	143.3	C1, C5, C3′
2′	H-2′	6.14 (1H, d, *J* = 10.3 Hz)	124.3	C4′, C6
3′	H-3′	-	206.8	
4′	H-4′		77.5	
5′	H-5′	1.45 (3H, s)	32.2	C3′, C4′, C5

Table S6. NMR Spectroscopic data of sordamentone A (6) (^{1}H at 500 MHz, ^{13}C at 125 MHz, Cl_3CD).

Position	Proton	δ_H (Hz)	$\delta^{13}C$	HMBC
1	H-1	-	125.3	
2	H-2	-	150.1	
3	H-3	6.68 (1H, dt, *J* =8.1, 0.7 Hz)	114.4	C1, C2, C5
4	H-4	7.16 (1H, dd, *J* =8.1, 7.4 Hz)	129.5	C2, C6
5	H-5	6.73 (1H, dt, *J* = 7.4, 0.7 Hz)	113.4	C1, C3, C6
6	H-6	-	143.1	
7	H-7a	5,15 (1H, dd, *J* = 12.3, 2.8 Hz)	70.8	C1, C2, C6, C1´,
	H-7b	5.07 (1H, dt, *J* = 12.3, 1.8 Hz)		C1,C2, C6 C1´,
1´	H-1´	5.72 (1H, q, *J* = 3.6, 2.8 Hz)	80.5	C6
2´	H-2a´	3.06 (1H, dd, *J* = 16, 7.8 Hz)	44.5	C1´, C3´, C6
	H-2b´	2.86 (1H, dd. *J* = 16, 4.7 Hz)		C1´, C3´, C6
3´	H-3´	-	210.5	
4´	H-4´	4.27 (1H, q, *J* = 7.2 Hz	73.4	C3´, C5´
5´	H-5´	1.41 (3H, d, *J* = 7.2 Hz)	19.3	C3´, C4´
C2-OH		4.94 (1H, s(br))		
C4´-OH		3.6 (1H, s(br))		

Table S7. NMR Spectroscopic data of sordamentone B (7) (^{1}H at 500 MHz, ^{13}C at 125 MHz, Cl_3CD).

Position	Proton	δ_H (Hz)	$\delta^{13}C$	HMBC
1	H-1	-	125.3	
2	H-2	-	150.1	
3	H-3	6.69 (1H, d(br), *J* =8.0 Hz)	114.4	C1, C2, C5
4	H-4	7.17 (1H, dd, *J* =8.0, 7.5 Hz)	129.5	C2, C6
5	H-5	6.75 (1H, d(br), *J* = 7.5 Hz)	113.4	C1, C3, C6
6	H-6	-	143.1	
7	H-7a	5,15 (1H, ddd, *J* = 12.3, 2.6, 1.1 Hz)	70.8	C1, C2, C6, C1´,
	H-7b	5.07 (1H, dt, *J* = 12.3, 2.6 Hz)		C1,C2, C6 C1´,
1´	H-1´	5.72 (1H, m)	80.5	C6
2´	H-2a´	2.96 (1H, dd, *J* = 16, 8 Hz)	44.5	C1´, C3´, C6
	H-2b´	2.91 (1H, dd. *J* = 16, 4.4 Hz)		C1´, C3´, C6
3´	H-3´	-	210.5	
4´	H-4´	4.30 (1H, q, *J* = 7.1 Hz	73.4	C3´, C5´
5´	H-5´	1.38 (3H, d, *J* = 7.1 Hz)	19.3	C3´, C4´
C2-OH		5.05 (1H, s(br))		
C4´-OH		3.6 (1H, s(br))		
